# Clinical characterization of epilepsy in children with chromosomal aberration 47, XXY

**DOI:** 10.1002/brb3.3178

**Published:** 2023-07-21

**Authors:** Congjie Chen, Yuanyuan Luo, Xueqing Hou, Tingsong Li

**Affiliations:** ^1^ Department of Rehabilitation Children's Hospital of Chongqing Medical University (CHCMU) Chongqing China; ^2^ Ministry of Education Key Laboratory of Child Development and Disorders Chongqing China; ^3^ National Clinical Research Center for Child Health and Disorders (Chongqing) Chongqing China; ^4^ International Science and Technology Cooperation Base of Child Development and Critical Disorders Chongqing China; ^5^ Chongqing Key Laboratory of Pediatrics Chongqing China; ^6^ Department of Neurology Children's Hospital of Chongqing Medical University (CHCMU) Chongqing China

**Keywords:** children, epilepsy, Klinefelter's syndrome, 47, XXY karyotype

## Abstract

**Objective:**

The phenotype of the chromosomal aberration 47, XXY exhibits considerable heterogenicity. In addition, epilepsy is extremely uncommon in individuals with this chromosomal disorder. As a result, the clinical characteristics of epilepsy in these patients remain poorly understood.

**Methods:**

Clinical data and the evolution of epilepsy in a boy diagnosed with chromosomal aberration 47, XXY were collected and analyzed. Furthermore, a systematic literature review was conducted to examine the relationship between chromosomal aberration 47, XXY and epilepsy in children.

**Results:**

We identified a novel phenotype associated with the chromosomal anomaly 47, XXY in a 2‐year‐2‐month‐old boy who presented with self‐limited epilepsy with autonomic seizures at onset, followed by developmental and/or epileptic encephalopathy with spike‐wave activation in sleep (D/EE‐SWAS), which was responsive to corticosteroid treatment. Including the present case, we analyzed 21 cases of children diagnosed with epilepsy due to the presence of the 47, XXY chromosomal anomaly. The most common types of epilepsy were focal combined generalized epilepsy (*n* = 9), epileptic spasms (*n* = 6), and generalized epilepsy (*n* = 4). There were six cases of infantile epileptic spasm syndrome (IESS) (*n* = 5) and developmental and epileptic encephalopathy (*n* = 1), one case of Lennox–Gastaut syndrome, and one case of D/EE‐SWAS. Apart from corticosteroids in IESS, 15 antiseizure medications (ASMs) were prescribed to eight children in this cohort, with valproate (*n* = 5) being the most frequently used.

**Conclusions:**

The epilepsy types and syndromes associated with the chromosomal anomaly 47, XXY demonstrated considerable heterogeneity. Among the observed phenotypes, IESS and focal epilepsy, which displayed partial responsiveness to multiple ASMs, were the most prevalent.

## Graphical abstract

The epilepsy phenotypes of chromosomal anomaly 47, XXY are largely heterogeneous. Focal epilepsy that partially responds to multiple ASMs is the most common phenotypes. Supernumerary X chromosome may exist in males with an X‐linked gene phenotype.

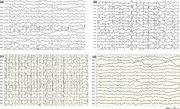

## 1 INTRODUCTION

Chromosomal aberration‐associated epilepsy is highly heterogeneous, ranging from self‐limited epilepsy (Guerrini, 2001; Wang et al., 2022) to refractory epilepsy (Kumada et al., 2005; Specchio et al., 2012). Klinefelter syndrome (KS) is the most common sex chromosome disorder in males, with an incidence of 0.02%−0.22% in individuals presumed male at birth. An estimated 87% of KS patients have the 47, XXY karyotype (Ridder et al., 2023). Although there are shared characteristics between KS and 47, XXY syndrome, considerable phenotypic differences also exist. KS is infrequently identified during childhood and adolescence, particularly in young boys, due to its largely normal or highly variable phenotype (Hopkins et al., 1979). Therefore, clinical data related to KS and 47, XXY chromosomal aberrations still need to be acquired and accumulated. In addition to hypogonadism, gynecomastia, and neuropsychological abnormalities, children with KS also occasionally present with epilepsy (Ferguson‐Smith, 1958). However, little is known about the epilepsy types and clinical evolution of cases harboring the 47, XXY chromosomal aberration, which is critical for optimizing diagnostic algorithm and antiseizure medications (ASMs).

In this study, we presented a case harboring 47, XXY karyotype that was unresponsive to multiple ASMs. We also summarized published data on the chromosomal abnormality 47, XXY implicated in childhood epilepsy to expand our current understanding of epilepsy associated with this genotype.

## 2 METHODS

In the retrospective study, we reviewed the medical charts of a KS‐diagnosed case and closely followed and assessed their clinical progression. Clinical data and laboratory tests included demographic data, seizure semiology and electroencephalographic (EEG) recordings, responsiveness to ASMs, neurological and endocrinological manifestations, and genetic tests.

We performed a systematic search of MEDLINE, China National Knowledge Infrastructure (CNKI), Embase, and Web of Science until October 2022 using “Klinefelter OR XXY” AND “Epilepsy OR Epileptic OR Seizure.” Articles not available in English or Chinese were excluded. We extracted and summarized the clinical data from the aforementioned published cases for patients under 18 years of age.

This study was approved by the Institutional Review Board of the Children's Hospital of Chongqing Medical of University (CHCMU, China). Written informed consent for the unpublished case was obtained from the patient's mother.

## 3 RESULTS

### 3.1 Case report

**FIGURE 1:**
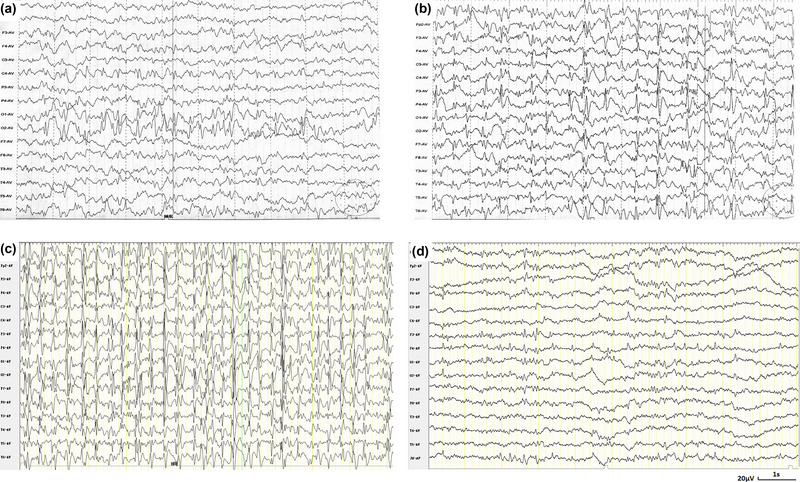
Electroencephalogram (EEG) evolution of case in this study. (a) Spike‐and‐wave discharge, predominantly in bilateral occipital areas at 2 years and 2 months of age. (b) 3−4‐Hz spike/poly‐spike‐and‐wave discharge, predominantly in parietal‐central‐posterior temporal region (3 years and 8 months old). (c) Pattern of continuous spikes during sleep at 4 years and 11 months of age, which improved significantly with corticosteroids and antiseizure medications (ASMs) (d). Scale bar: 20 μV/s.

At the age of 2 years and 2 months, a previously developmentally normal boy experienced a unilateral tonic seizure lasting approximately 20 min after a minor head injury, without fever. The boy was born full‐term to non‐consanguineous parents after a normal pregnancy, and family history of seizures was unremarkable. Interictal EEG displayed a slow background and dominant spike‐waves in the temporal‐occipital area (Figure 1a). At the initial presentation, magnetic resonance imaging (MRI) of the brain revealed normal results, except for an enlarged cavum septum pellucidum. At this stage, no ASMs were introduced. However, 1 month later, a focal tonic seizure following pallor and vomiting occurred, lasting approximately 20 min while in a drowsy state. Thereafter, oxcarbazepine (OXC) was initiated according to the seizure type and suspected self‐limited epilepsy with autonomic seizures. However, seizure recurred monthly during sleep over the following 18 months. Levetiracetam (LEV) and clonazepam (CZP) were trialed sequentially. Seizure‐freedom was achieved and maintained for 8 months on LEV (42 mg/kg/day) and CZP (0.1 mg/kg/day). However, at 3 years and 8 months of age, the patient developed apparently generalized tonic‐clonic seizures and was subsequently administered valproate (VPA). Seizure‐freedom persisted for more than 2 years with VPA (17 mg/kg/day), LEV (37 mg/kg/day), and NZP (0.3 mg/kg/day, due to the inaccessibility of CZP). However, EEG evolution was remarkable, from predominantly interictal occipital discharge to developmental and/or epileptic encephalopathy with spike‐wave activation in sleep (D/EE‐SWAS) at 4 years 11 months (Figure 1c). Consequently, the patient was administered intravenous methylprednisolone (16 mg/kg/day) for 3 consecutive days, followed by oral prednisone (2 mg/kg/day). Electrical status epilepticus in sleep on EEG was significantly improved 2 months later with prednisone (1.25 mg/kg/day), LEV (35 mg/kg/day), NZP (0.3 mg/kg/day), and VPA (12 mg/kg/day) (Figure 1d).

At 5 years and 8 months, his parents noted suspected intellectual disability. His tested intelligence quotient (IQ) score was 53, and S‐M score was borderline. According to the reported genetic variants associated with D/EE‐SWAS (Specchio et al., 2022), whole‐exome sequencing and copy number variation analysis were performed, yielding an XXY karyotype without any other pathological variants, as further confirmed by chromosomal karyotyping. A provisional diagnosis of KS was established, and the case was referred to a pediatric endocrinologist. Of note, KS‐associated endocrinology tests, including serum levels of adrenocorticotropic hormone, cortisol, thyroxine, androstenedione, dehydroepiandrosterone, α‐hydroxyprogesterone, insulin, follicle‐stimulating hormone, luteinizing hormone, progesterone, prolactin, estradiol, total testosterone, and sex hormone‐binding globulin, were all unremarkable.

### 3.2 Literature search results

**FIGURE 2:**
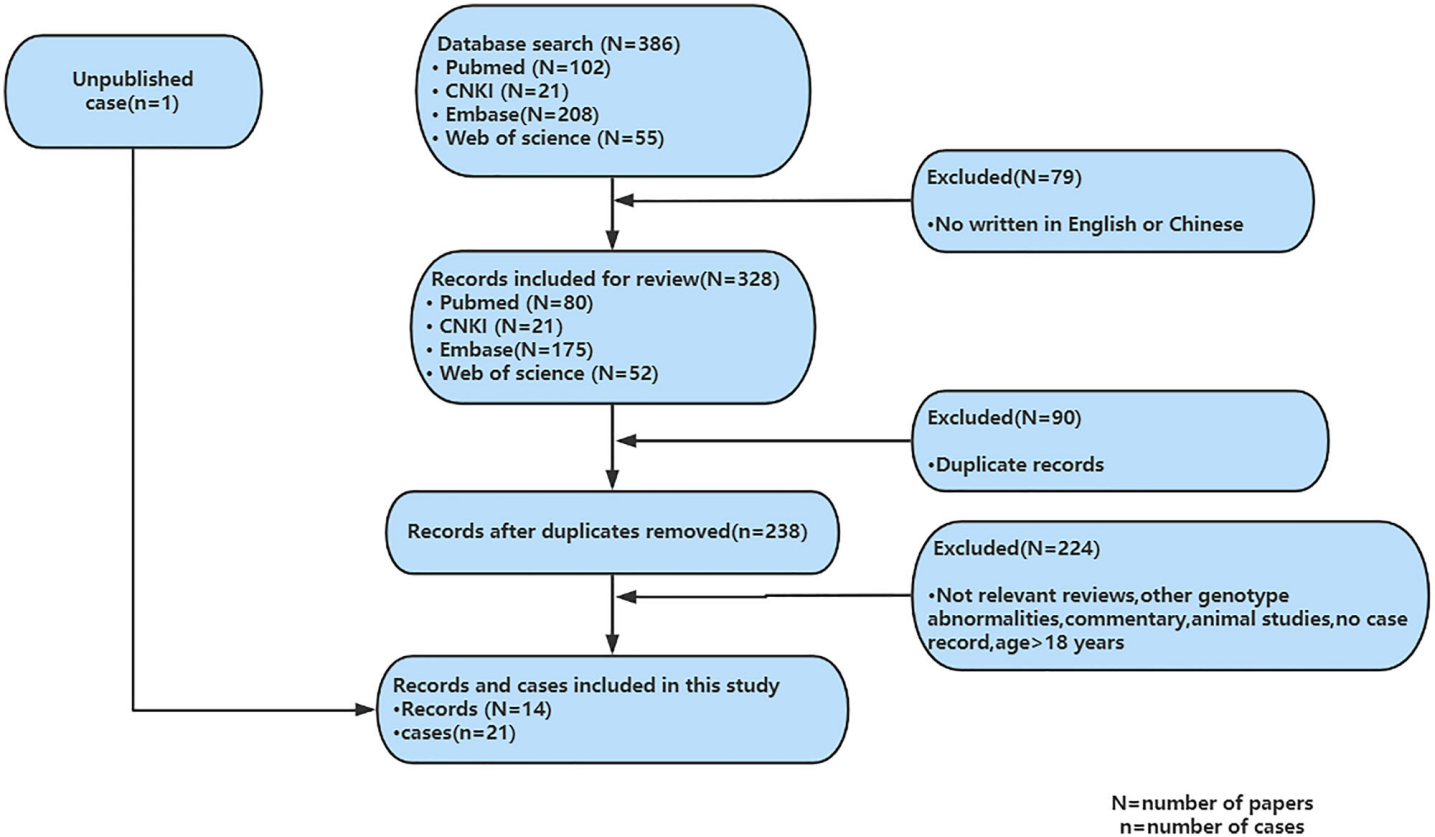
Algorithm of systematic review.

As shown in Figure 2, 386 citations were retrieved from four databases. A combination of manual and citation manager deduplication was used to eliminate 90 duplicate citations and an additional 79 citations published in neither English nor Chinese, yielding a total of 238 citations for review. After abstract‐level elimination and full manual review, a total of 14 papers reporting detailed clinical data for 20 patients with 47, XXY were identified and included. All included studies were descriptive and retrospective. Detailed data on the 20 published cases are provided in Table 1.

**TABLE 1:** Clinical features of enrolled cases.

Patient onset age	Seizure semiology	EEG	Brain imaging	Treatment	Neurological examination	Seizure free
1 (2 M) (Brunetti et al., 2018)	GTC, spasm, DEE	Multifocal discharge	Multiple cerebral infarctions	PHT + PB + CBZ	Severe MR	No
2 (6 M) (Kim et al., 2014)	GTC, CPS	L or R F SW	Ventriculomegaly	VPA	MR	Yes
3 (8 M) (Elia et al., 1995)	Febrile Szs, Focal Szs	Posterior regions spikes and SW	Normal	PB + CBZ	MR	Yes
4 (22 M) (Tatum et al., 1998)	CPS, GTC	Abnormal background, multifocal spikes	Normal	N/A	IQ 75	No
5 (2 Y) (Elia et al., 1995)	GTC	Normal	Ventriculomegaly	N/A	MR, hypotonia, HETR	Yes
6 (2 Y) (Zhiyi et al., 2019)	LGS	SB; 1.5−2.5 Hz GSW and fast rhythm	Moderate diffuse atrophy	CLB + LTG + rufinamide + perampanel + VPA, Surgery	MR	No
7 (3 Y) (Grosso et al., 2004)	CPS	Bioccipital spikes	Normal	N/A	N/A	Yes
8 (5 Y) (Boltshauser et al., 1978)	Petit mal, grand mal	SB, paroxysmal discharges	Ventriculomegaly	ASMS (N/A)	MR, BP	Yes
9 (7 Y) (Boltshauser et al., 1978)	Typical ABS	Typical 3 Hz SW	N/A	Succinimide→VPA	Normal IQ, BP	Yes
10 (7 Y) (Miyamoto et al., 1992)	GTC	SB, SW	Normal	CBZ + PHT + primidone + ZON	MR	Yes
11 (8 Y) (Gavaret et al., 1997)	ABS, GTC	Typical generalized 3 Hz SW	Cortico‐subcortical atrophy	PB + VPA + ethosuximide	IQ80, psychotic personality	Yes
12 (9 Y) (Grosso et al., 2004)	CPS, GTC	Multifocal spikes	Normal	N/A	N/A	Yes
13 (12 Y) (Tatum et al., 1998)	ABS, GTC	Multifocal spikes	Normal	N/A	IQ 70	No
14 (14 Y) (Tatum et al., 1998)	GTC	Abnormal background, R T spikes	Normal	N/A	Mild MR	Yes
15 (17 Y) (Tatum et al., 1998)	GTC	Abnormal background, Cz Polyspikes	Punctate white matter lesions	N/A	Normal IQ	No
16 (26 M)^a^	DEE‐SWAS	O P sharp‐and‐wave ESES (SWI > 90%)	Normal	OXC→VPA + LEV + NZP, methylprednisolone	IQ 53	Yes
17 (4 M) (Inoue et al., 2012)	IESS	Hypsarrhythmia→ R PT focal spikes	Normal	ACTH + VPA + NZP	Normal IQ	Yes
18 (1 D) (Chen et al., 2009)	IESS	Paroxysmal sharp‐and‐wave complexes	Lissencephaly, partial ACC, ventriculomegaly	VGB + NZP + OXC	MR, MC, hypotonia	N/A
19 (2 M) (Shetty et al., 2014)	IESS	Hypsarrhythmia→Resolution of the hypsarrhythmia	Colpocephaly, ACC, ventriculomegaly	Prednisolone + VGB	MR, MC	Yes
20 (2 M) (Zubairi et al., 2009)	IESS	Bilateral asynchronous sharp‐and‐wave	Colpocephaly, ACC	PB + PHT + CBZ + VPA + NZP + CZP + CLB OR TPM + VGB + ZON	MR	No
21 (2 M) (Hopkins et al., 1979)	IESS, focal Szs	Bilateral asynchronous spike, sharp and slow wave	ACC, ventriculomegaly	N/A	MR, MC	N/A

Abbreviations: ABS, absence;ACC, agenesis of corpus callosum;ACTH, adrenocorticotropic hormone;ASM, anti‐seizure medication;BP, behavioral problems;C, central;CBZ, carbamazepine;CLB, clobazam;CPS, complex partial seizure;CZP, clonazepam;D, days;DEE, developmental epileptic encephalopathy;DEE‐SWAS, developmental epileptic encephalopathy with spike‐and‐wave activation in sleep;EEG, electroencephalogram;ESES, electrical status epilepticus during sleep;F, frontal;FA, facial anomalies: telecanthus, upslanting eyelids, thin lips;GSW, generalized spike‐and‐wave;GTC, generalized tonic‐clonic;HETR, hyperexcitable tendon reflexes;IESS, infantile epileptic spasms syndrome;IQ, intelligence quotient;L, left;LTG, lamotrigine;M, months;MC, microcephaly;MR, mental retardation;N/A, not available;NZP, nitrazepam;O, occipital;OXC, oxcarbazepine;P, parietal;PB, phenobarbital;PHT, phenytoin;PSW, polyspike‐and‐wave;PT, posterotemporal;R, right;SB, slow background;SW, spike‐and‐wave;SWI, spike‐wave index;Sz, seizure;T, temporal;VGB, vigabatrin;VPA, valproate;Y, years;ZON, zonisamide;→, EEG from onset to follow up.

^a^ Current case.

A total of 21 patients were enrolled in the present study, comprising the published studies and the unpublished case reported here.

### 3.3 Phenotypic analysis

Of the 21 cases harboring the 47, XXY karyotype and presenting with epilepsy, all were phenotypically male. As shown in Table 1, five cases (23.8%) were diagnosed with infantile epileptic spasm syndrome (IESS). Among these cases, two boys (cases 19 and 21) met the diagnostic criteria for Aicardi syndrome, and two boys (cases 18 and 20) were classified as suggestive cases phenotypically (Wong & Sutton, 2018). Thus, 17 cases were diagnosed or suspected with KS.

Among the 17 cases of KS, the median age of onset was 3 years (2 months to 17 years). For cases with data regarding neurological examination, 12 of 15 patients had varying degrees of mental retardation and/or behavioral problems (cases 1−6, 8, 10−11, 13−14, 16). Hypotonia and hyperexcitable tendon reflex was reported in case 5. For the KS cases, only one individual (case 3) had a history of febrile seizure.

For cases with endocrinological screening (cases 9−11), all presented with a feminine appearance, with a median onset age of 30 (7−36 years). Other symptoms included testicular atrophy (*n* = 3), soft and delicate skin (*n* = 2), gynecomastia (*n* = 1), feminine fat distribution (*n* = 1), and sparse truncal hair (*n* = 1).

Brain imaging was performed in 20 cases, with eight individuals exhibiting non‐specific abnormal findings, including ventricular dilatation (*n* = 6, cases 2, 5, 8, 18, 19, and 21), agenesis of corpus callosum (*n* = 4, cases 18−21), colpocephaly (*n* = 2, cases 19 and 20), lissencephaly (*n* = 1, case 18), brain atrophy (*n* = 2, cases 6 and 11), punctate white matter lesions (*n* = 1, case 15), and multiple cerebral infarctions (*n* = 1, case 1).

### 3.4 Epilepsy and ASMs

In regard to epilepsy type, nine cases were diagnosed with focal combined generalized epilepsy (cases 2−3, 4, 6, 7, 10, 12, 14, and 16), six cases were diagnosed with epileptic spasms (cases 1 and 17−21), four cases were diagnosed with generalized epilepsy (cases 5, 8, 9, and 11), and two cases were diagnosed with unknown type (cases 13 and 15). For the epilepsy syndromes, there were five cases of IESS (cases 17−21), two cases of typical absence epilepsy (cases 8 and 9), one case of developmental and epileptic encephalopathy (case 1), one case of Lennox–Gastaut syndrome (case 6), and one case of D/EE‐SWAS (case 16). The remaining 11 cases were classified as non‐syndromic epilepsy. A total of 15 ASMs were introduced in a cohort of eight cases (cases 1−3, 6, 9−11, and 16), excluding IESS and cases for which no ASM details were available. Among them, OXC and succinimide were eventually replaced by other ASMs. VPA was the most frequently used ASM (*n* = 5), followed by phenobarbital (*n* = 3) and carbamazepine (*n* = 3). At the end of follow‐up, except for cases 2 and 9 who were seizure‐free after VPA monotherapy, the other six cases were treated with two or more ASMs.

Regarding the EEG findings, all 17 patients had interictal EEG recordings, except for the five IESS cases. Three cases showed generalized discharge (cases 8, 9, and 11), while four cases showed spikes predominantly in posterior regions, including the temporal, central, parietal, and occipital areas (cases 3, 7, 14, and 16).

## 4 DISCUSSION

Our current case expanded the phenotype of 47, XXY abnormality with D/EE‐SWAS, which was responsive to corticosteroids. Furthermore, we also elucidated the clinical characteristics of this chromosomal disorder, particularly with respect to epilepsy. Apart from IESS, focal epilepsy and focal combined generalized epilepsy were the most frequently observed epilepsy types in individuals with KS. Most of these cases were non‐syndromic and exhibited varying degrees of intellectual disability.

To the best of our knowledge, this study is the first to report on a case of 47, XXY chromosomal aberration with D/EE‐SWAS. Interestingly, this patient initially presented with EEG features of self‐limited epilepsy with autonomic seizures. However, due to the unresponsive nature of the individual to multiple ASMs, progressive deterioration in EEG findings, and the absence of acquired etiology, a genetic factor was suspected. Therefore, although chromosomal aberrations are rarely associated with D/EE‐SWAS, karyotypic analysis for 47, XXY should be considered and performed when necessary.

The estimated incidence of epilepsy linked to the 47, XXY chromosomal abnormality is ∼5.5% (Nieschlag, 2013). However, the specific types and syndromes of epilepsy associated with this disorder have yet to be fully documented and explored, primarily due to its rarity. The present study revealed that focal‐onset seizures were the most prevalent seizure type within this cohort, followed by generalized epilepsy, with occasional myoclonic, absence, and atonic seizures also reported. Among the epilepsy syndromes observed, IESS accounted for five cases (20.0%), followed by developmental and epileptic encephalopathy (*n* = 1). Given the extensive phenotypic heterogeneity associated with this abnormality, ASM selection was primarily based on specific epilepsy type. However, as other chromosomal abnormalities are associated with epilepsy (Singh et al., 2002), the considerable heterogeneity of epilepsy type makes it challenging to establish definitive conclusions and guidelines regarding when karyotyping should be performed or how to optimize treatment strategies. To determine the underlying etiology of epilepsy, it is crucial to incorporate chromosomal analysis, particularly in cases of IESS and partially responsive focal or apparent generalized epilepsy, especially when considering genetic or “unknown” causes. Additionally, given the presence of multiple epilepsy types, VPA should potentially be considered a first‐line treatment, as indicated by the findings in our study cohort.

While testosterone deficiency, small and firm testes, and infertility are considered the primary symptoms of 47, XXY, there are no reliable indicators of KS in prepubertal children (Nieschlag, 2013). Indeed, apart from epilepsy and lower IQ, no endocrinological tests yielded positive results in our case, nor have they been reported in previously published cases, except for three patients who exhibited symptoms as early as 7 years of age.

Unexpectedly, two patients have been reported to carry de novo *CDKL5* (Sartori et al., 2009) and de novo *PCDH19* (Romasko et al., 2018), both located on the X chromosome. These two cases present with a *CDKL5* and *PCDH19* phenotype, respectively, rather than the common features of KS in terms of onset age, epilepsy type, and hypogonadism. Aicardi syndrome, which primarily affects females, has also occasionally been reported in male cases with 47, XXY chromosomal aberrations (Chen et al., 2009; Hopkins et al., 1979; Kroner et al., 2008; Shetty et al., 2014; Zubairi et al., 2009). It is hypothesized that the presence of two X‐chromosomes may have a beneficial effect, with a balanced pattern of inactivation between the two neuronal populations (Depienne & LeGuern, 2012). These findings suggest that karyotyping of the supernumerary X chromosome may be warranted when an X‐linked gene phenotype is present in males and chromosomal anomalies co‐exist within an individual.

The underlying mechanisms associated with the 47, XXY epileptic phenotype are yet to be fully understood. Alterations in brain structure and activation of the amygdala as well as epigenetics have been found in these patients (Skakkebæk et al., 2021). However, the exact epileptogenic mechanism underlying this aberration is still unclear. Previous research has shown that the impact of 15q 13.3 microdeletion on transcriptome dysregulation during brain development is ubiquitous rather than specific (Körner et al., 2022). Therefore, it can be hypothesized that the “triplo‐excess” of genes in 47, XXY can lead to non‐specific neurological abnormalities, such as mental retardation and epilepsy, due to its impact on neuronal development, migration, differentiation, and functioning (Skakkebæk et al., 2021). However, the rarity and heterogeneity of epilepsy in KS, along with the co‐existence of the 47, XXY karyotype and other genetic variants in epilepsy, suggest that the 47, XXY karyotype may only be an incidental finding in some cases of epilepsy, rather than a causative genotype.

The main limitations of this research are the sample size of enrolled patients and the retrospective nature of the study, which make it difficult to gain a comprehensive understanding of this disorder, particularly long‐term prognosis, endocrinological disturbances, and responsiveness to ASMs. Hence, long‐term and multi‐center prospective studies are necessary to clarify the aforementioned concerns.

Collectively, the epileptic phenotypes of the 47, XXY aberration are highly heterogeneous. Apart from IESS, the predominant epilepsy type was focal‐onset epilepsy. In cases of ASM‐unresponsive epilepsy with an “unknown” cause, karyotyping and chromosomal microarray analysis should be considered, particularly after conducting whole‐exome sequencing and other relevant tests and especially for males displaying X‐linked gene phenotypes.

### PEER REVIEW

The peer review history for this article is available at https://publons.com/publon/10.1002/brb3.3178.

## Data Availability

All the data of this study can be reached upon the qualified request.
